# Double Rabi splitting in methylene blue dye-Ag nanocavity

**DOI:** 10.1515/nanoph-2021-0697

**Published:** 2022-01-04

**Authors:** Xiaobo Han, Fang Li, Zhicong He, Yahui Liu, Huatian Hu, Kai Wang, Peixiang Lu

**Affiliations:** Hubei Key Laboratory of Optical Information and Pattern Recognition, Wuhan Institute of Technology, Wuhan 430205, China; Wuhan National Laboratory for Optoelectronics and School of Physics, Huazhong University of Science and Technology, Wuhan 430074, China; School of Mechanical and Electrical Engineering, School of Optical Information and Energy Engineering, Wuhan Institute of Technology, Wuhan 430205, China; Guangdong Intelligent Robotics Institute, Dongguan 523808, China

**Keywords:** dimer exciton, double Rabi splitting, methylene blue, plasmonic nanocavity, strong coupling

## Abstract

We demonstrate a double Rabi splitting totaling 348 meV in an Ag nanocavity embedding of methylene blue (MB) dye layer, which is ascribed to the equilibrium state of monomer and dimer coexistence in MB dye. At low dye concentration, the single-mode strong coupling between the monomer exciton in MB dye and the Ag nanocavity is observed. As the dye concentration is increased, three hybridized plexciton states are observed, indicating a double Rabi splitting (178 and 170 meV). Furthermore, the double anti-crossing behavior of the three hybrid states is observed by tuning the Ag nanocube size, which validates the multi-mode strong coupling regime. It shows clear evidence on the diverse exciton forms of dye molecules, both of which can interact with plasmonic nanocavity, effectively. Therefore, it provides a good candidate for realizing the multi-mode strong coupling.

Recently, the hybrid systems of emitters and cavities have attracted great interest, because the optical response of emitters can be modulated or give rise to novel quantum states depending on the coupling strength, *g* [1], [2], [3], [4], [5], [6]. Under the week coupling condition, the coupling strength is such that the energy exchange from the emitter to the cavity occurs but is nonreversible and incoherent. Typically, this regime has been used to increase the spontaneous emission of emitters by the Purcell effect [7], [8], [9], [10]. Under the strong coupling condition, the coupling strength between the emitter and cavity is so large that the emitter and the cavity become a new single quantum object with energy shared coherently and reversibly between them [11], [12], [13], [14], [15]. When the coupling strength is high enough, the ultra-strong coupling regime will be reached [16], [17], [18]. Therefore, it is essential to enlarge the coupling strength between exciton and nanocavity. Since the coupling strength is positively correlated with exciton numbers ( $g\propto \sqrt{N}$ ), it can usually be enhanced by increasing the exciton numbers (*N*) involved in the coupling [19], [20], [21]. Among kinds of emitters for strong coupling, dye molecules show many advantages, such as unusually large transition dipole moment, controllable molecular orientation and high concentration [21], [22], [23], [24]. And it is often used to enhance the coupling strength by increasing the dye concentration in plasmon–exciton strong coupling [25]. However, with the increase of dye concentration, new exciton states may be generated by the interaction between dye molecules (such as dimer exciton), which has an impact effect on strong coupling phenomena, such as the formation of multi-mode coupling.

Multi-mode strong coupling in hybrid excitonic systems is a very interesting extension of single-mode strong coupling, because they integrate numbers of qubits in which quantum states can be efficiently manipulated. It leads to a large variety of scientific and technological frontiers, such as more energy dissipation channels and greater modulation [26], [27], [28], [29], [30], [31], [32], [33], [34]. The possibility to induce multi-mode coherent hybrid states can envision an interesting perspective for a better comprehension of the strong coupling dynamics, such as the energy transfer between multiple exciton-SPP branches [31, 32]. With multiple modes participating in the coupling, the coverage frequency range is significantly expanded [30]. Moreover, multi-mode strong coupling can open the way to multipartite entanglement and the implementation of quantum computing devices using multi-quasiparticle boson systems [35]. The emergence of multiple Rabi splitting in these systems is of considerable interest and relevance for both fundamental and applied science, providing remarkable opportunities for studying multimode hybridization and energy transfer [32], [33], [34, 36, 37].

The previous works on the multi-mode strong coupling are based on dye-microcavity system, however, it has few reported in the single dye-plasmonic nanocavity system. Since the plasmonic nanocavity can concentrate light energy into nanoscales, leading to a strong local-field in an ultrasmall mode volume [2]. Compared with the microcavity, the nanocavity usually has a wide resonance range, which is more suitable for simultaneous coupling of different excitons with larger energy differences. However, it is still challenging to achieve multi-mode strong coupling between excitons and plasmonic nanocavity, because of the high intrinsic loss of metal, low cooperativity and quality factor in comparison with those of microcavity. Therefore, it is necessary to design a hybrid nanostructure containing two series of excitons with large energy difference, which spectrally matches the surface plasmon polariton in nanocavity.

In this letter, we report for the first time on the observation of the multi-mode strong coupling in an Ag nanocavity containing methylene blue (MB) dye layer under ambient conditions. It is ascribed to the collective interaction between the monomer, dimer excitons in concentrated MB dyes (10^−6^ to 10^−3^ M) and plasmon. At a low dye concentration of 1 × 10^−6^ M, strong coupling between monomer exciton and localized surface plasmon is achieved, leading to a Rabi splitting up to 147 meV. As increasing the dye concentration to 1 × 10^−4^ M, the three new hybridized states appear in similar hybrids, leading to a double Rabi splitting of 178 and 170 meV. Double anti-crossing behavior is further presented by changing the size of Ag nanocubes, which confirms the strong coupling mechanism. The experiment demonstrates the dimer exciton influence on the strong coupling behavior. Furthermore, the strong coupling between dimer excitons, monomer excitons and plasmons can be actively reversible manipulated by tuning the temperature.

## Results and discussion

**Figure 1:**
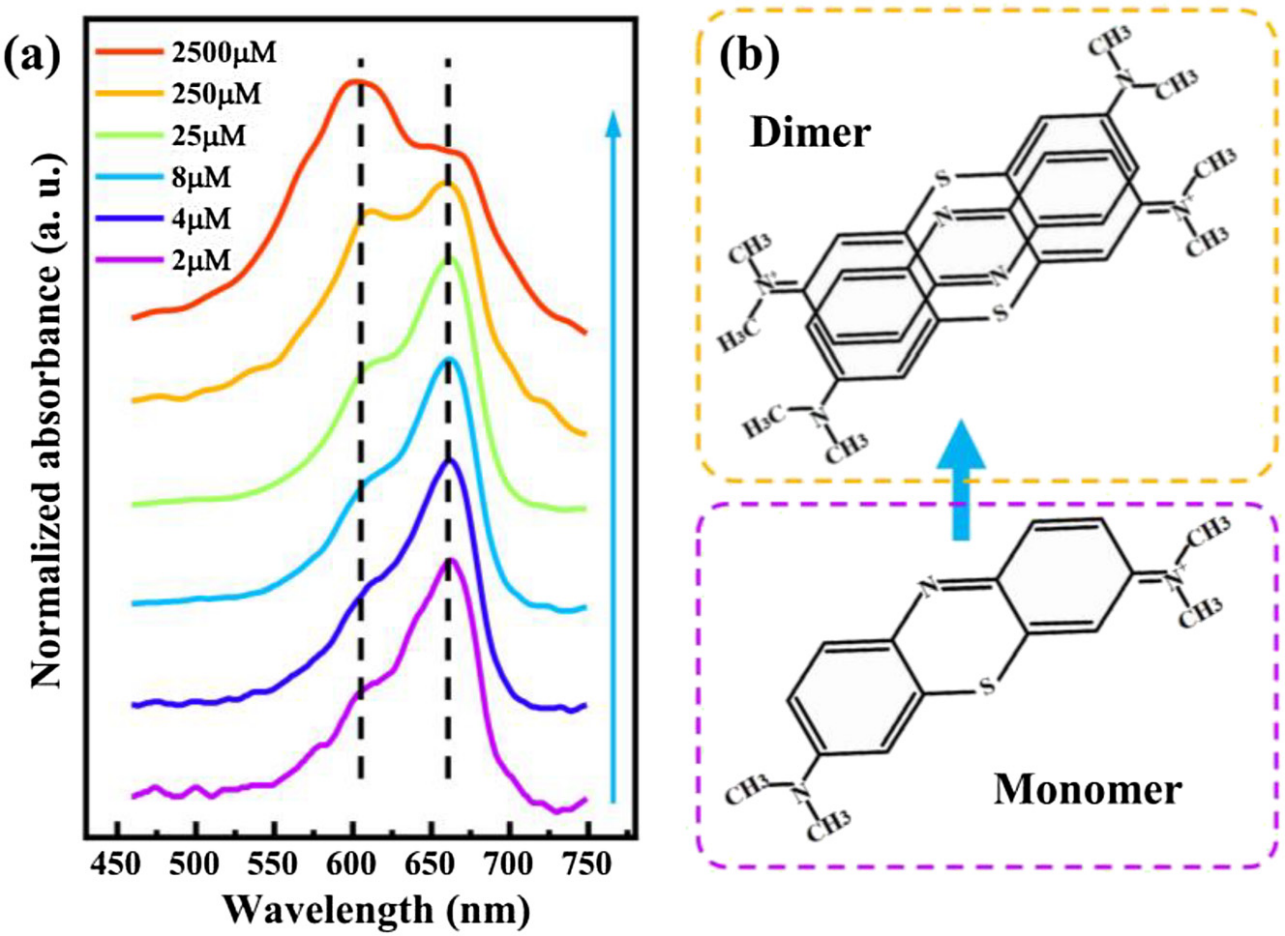
Absorption of MB monomer and dimer molecules. (a) UV–Vis absorption spectra for MB dye molecules dispersed in PVA polymer spin-coated on coverslips at different concentrations. The two absorption peaks respectively represent the absorption of MB monomer and dimer molecules. Dashed lines indicate the positions of the two absorption peaks. The blue arrow indicates increasing dye concentration. (b) Chemical structure diagram showing the monomer and probable dimer of MB.

To investigate the interaction between exciton and plasmon, MB dye is chosen, which is a common candidate dye for strong coupling [21]. The MB dye used in the present study exists in monomeric as well as aggregated forms with distinct spectral features [38]. Aggregation is one of the features of dyes in solution, affecting their photo-physical properties. In the concentration range from 10^−6^ to 10^−3^ M, the extent of aggregation of MB is limited to dimerization. UV–Vis absorption spectroscopy is one of the most suitable methods for quantitatively studying the aggregation phenomena of dyes as a function of concentration [39]. In order to form stable and homogeneous dye film, MB is doped polyvinyl alcohol (PVA), which is an ordinary dielectric material. Then, PVA-MB aqueous solution is spin-coated onto coverslips. In Figure 1a, normalized UV–Vis absorption spectra of the PVA-MB layer are shown at different concentrations. It can be seen that all spectra appear both the absorption band at around 610 and 665 nm, corresponding to the absorption intensity of dimer and monomer of MB dye, respectively [38]. At a low MB concentration, for example, 2 μM shown in Figure 1a (purple curve), the absorption spectrum shows a broad absorbance band at around 665 nm, and a weak absorbance band at about 610 nm. It demonstrates that nearly all MB molecules are in the monomer form at the concentration of 2 μM. As the MB concentration increased, the absorbance band near 610 nm becomes stronger, indicating that more dimers were formed. At the high concentration (2.5 mM), the absorption intensity of dimer is even stronger than the monomer one. Also, it indicates equilibrium between two spectral species, i.e., monomer–dimer equilibrium. Figure 1b shows the chemical structure of MB monomer, and the probable chemical structure of MB dimer. The MB dimerization equilibrium can be expressed as 2(Dye)_mon_ ↔ (Dye)_dim_. Thus, the presence of dimer at various concentration would considerably influence the strong coupling behavior between dye and plasmonic nanostructures.

**Figure 2:**
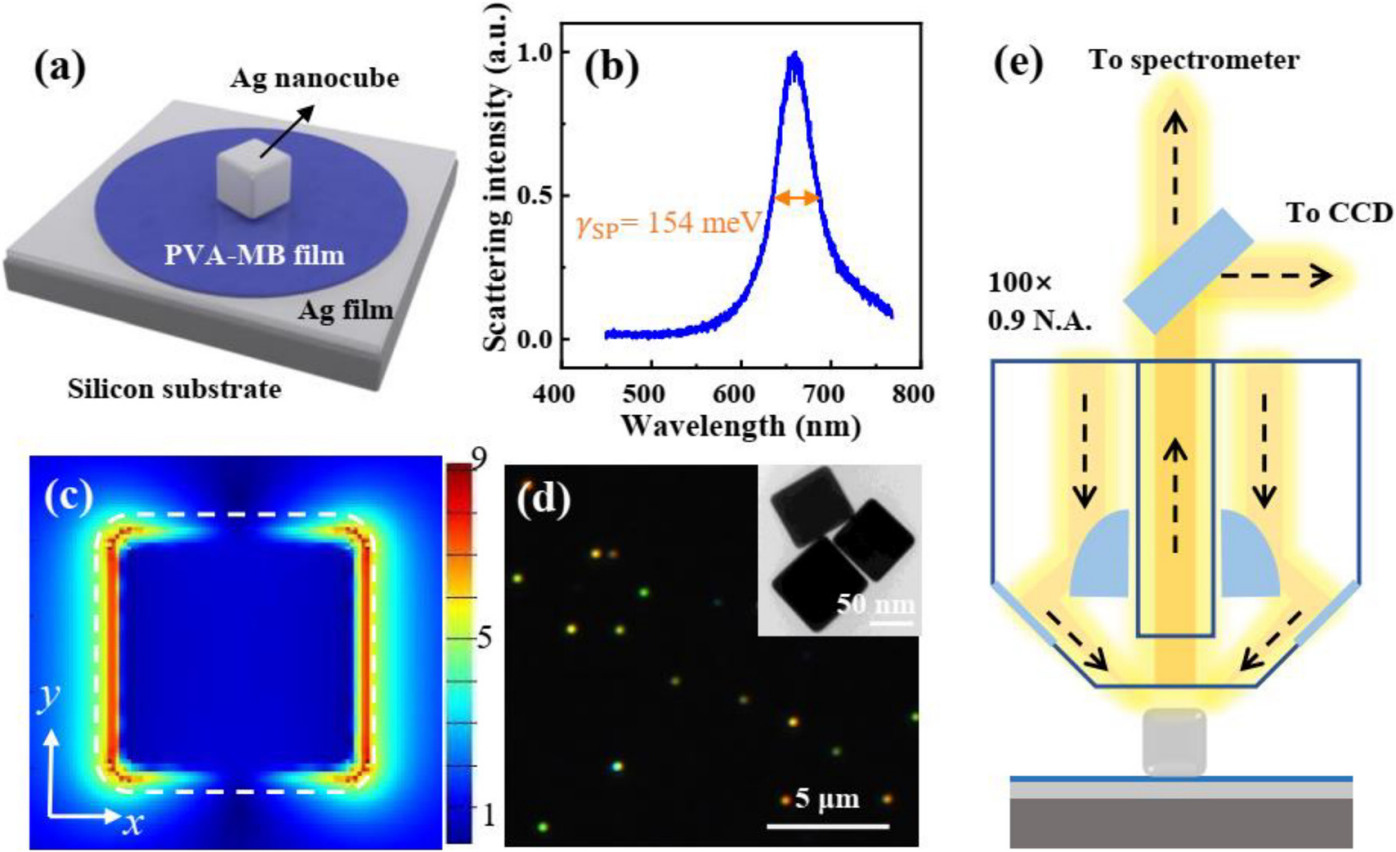
Optical characterization of the MB dye-Ag nanocavity. (a) Schematic representation of a hybrid nanostructure, made of an Ag film, a PVA film doped with MB dye molecules, and an Ag nanocube on a silicon substrate. (b) Normalized scattering spectrum of an Ag nanocavity without dye molecules. (c) Electric field distribution in *xy* plane of the plasmonic nanocavity calculated by finite-difference time-domain method (FDTD). The incident light is polarized along the *x*-axis. White dashed lines indicate the outline of the Ag nanocube. (d) Dark-field microscope image of the hybrid nanostructures. Bright spots indicate individual Ag nanocubes. Inset: TEM image of Ag nanocubes. (e) Experimental apparatus for measuring the dark-field scattering signal of a single nanoparticle. The sample is obliquely irradiated by the white light and the vertical scattering signal is collected by a BD objective.

For realizing the strong coupling between excitons from MB dye and surface plasmons, the plasmonic nanocavity in a nanoparticle-on-mirror (NPoM) system is chosen for the design of hybrid nanostructures, which consists of a single Ag nanocube and an Ag film, as illustrated in Figure 2a. It is a well-known plasmonic system with broad tunability [7, 21, 23, 40]. The gap between the nanocube and the Ag film is tuned by the thickness of PVA. Thus, the surface plasmon resonance (SPR) can be adjusted by the gap thickness. Figure 2b shows the scattering spectrum of the Ag nanocavity, which is tuned to overlap the absorption peak of MB molecule (spectra of different nanocube sizes, see Figure S1). The plasmon line width of the nanocavity $\gamma_{\text{SP}}$ can be extracted to be ∼154 meV. Figure 2c shows the distribution of simulated electric field in the *xy*-plane at resonance in the plasmonic nanocavity, which is a similar dipole oscillation along the nanocube edge. The sub-10 nm vertical dimension of the nanocavity results in a large electric-field enhancement, which is helpful to achieve the strong coupling mechanism. We then replace the pure PVA layer with the MB doped PVA layer to introduce the excitons. Briefly, PVA-MB layer is spin-coated onto the Ag film, followed by drop-casting Ag nanocubes to form the hybrid nanostructure (see sample preparation for details). Figure 2d shows a dark-field microscope image of the hybrids. Brightly colorful spots indicate the individually separate Ag nanocubes. Different colors represent the different sizes of Ag nanocubes, which is related to the SPR position. The transmission electron microscopy (TEM) image in the inset shows different Ag nanocubes, indicating the square shape. To characterize strong coupling for individual nanostructures, previous works show that single-particle dark-field scattering measurement is convenient [21, 23, 24, 40], [41], [42]. Figure 1e illustrates the dark-field scattering experimental setup for individual nanoparticles. The samples were irradiated by a dark-field objective (Olympus, 100×, NA = 0.9, MPLFLN), and the scattered light was collected by the same objective. The signal is then sent to a CCD (Qimaging, QICAM B series) or a spectrometer (Andor, 303i). All the experiments were done at room temperature.

**Figure 3:**
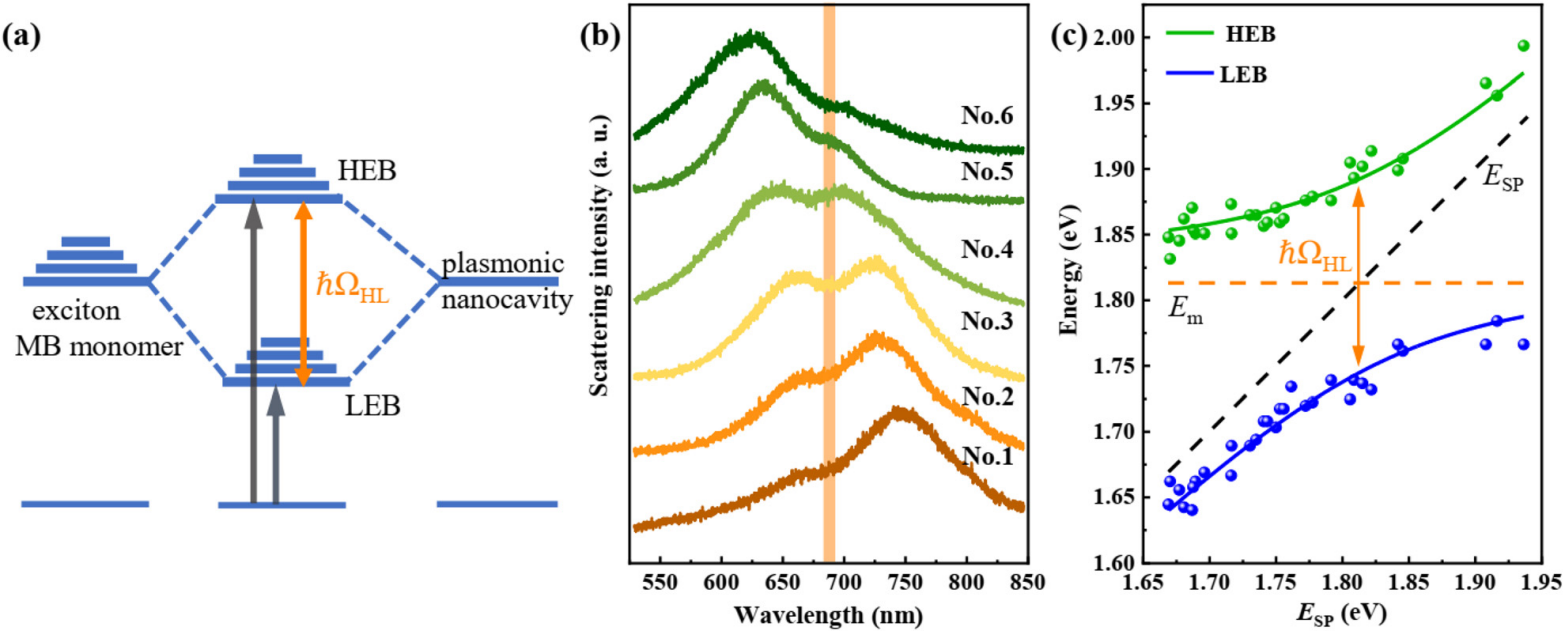
Strong monomer exciton-plasmon coupling. (a) Schematic representation of the strong coupling between the exciton of the monomeric dye molecule (left) and the surface plasmon of nanocavity (right), leading to the formation of two new eigen hybrid light–matter states: the high energy branch (HEB) and the low one (LEB). The Rabi splitting energy is marked as ℏΩ_HL_. (b) Scattering spectra of the hybrid nanostructures with different Ag nanocube sizes (sample numbers from No. 1 to No. 6). All spectra appear two peaks and a dip at the exciton resonance of MB. SPR wavelength of the plasmonic nanocavity is tailored across the exciton resonance of MB. (c) Dispersion of plexciton with HEB and LEB varied as a function of plasmon energy. Green and blue dots indicate experimental data extracted from scattering spectra. The solid lines are fitting results by the coupled harmonic oscillator model. The black dash line presents the plasmon energy of the pure plasmonic nanocavity, while the orange dash line indicates the exciton resonance of MB. Orange arrow marks the Rabi splitting energy of the hybrid nanostructures, giving a splitting of ℏΩ_HL_ = 147 meV.

We investigated the optical properties of the nanocavity with MB at a low concentration (1 × 10^−6^ M) at first. Due to the absorbance of monomer is much stronger than that of the dimer, optical transitions in this common type of hybrid system are schematically illustrated in Figure 3a. The hybrid states are formed when energies are exchanged between exciton of MB monomer and plasmons of a nanocavity at a rate faster than their respective dephasing processes. This back-and-forth exchange, Rabi oscillation, leads to a quantum superposition of the uncoupled molecular transition and SPR. It results in the generation of two new eigenstates for the system, the high energy branch (HEB) and the low one (LEB) of plexcitonic states, separated by the Rabi-splitting ℏΩ_HL_. In Figure 3b, the scattering spectra of hybrid nanostructures with different Ag nanocube sizes are measured (see more spectra in Figure S2). All normalized scattering spectra show two peaks and a dip at ∼680 nm, which is the exciton resonance of MB monomer. It is redshifted from 665 to 680 nm, which may be caused by the increased temperature in the nanogap due to the efficient dye absorption under the plasmonic resonance [43]. The scattering peaks are progressively tuned across the monomeric absorbance band. In the yellow curve, the splitting appears a deeper dip and the line shape is almost symmetric, indicating the plasmon mode overlaps well with the MB monomeric exciton.

To map the dispersion curve, we extract the two peaks of multiple scattering spectra, as shown in Figure 3c (green and blue dots). The dashed lines with indicators show the uncoupled plasmon and monomer exciton modes. The traces of all peaks present an anti-crossing curve. The anti-crossing behavior is a typical characteristic of the strong coupling between exciton and plasmon. The whole system can be simply described by the two-coupled oscillator:

(1) $$\left(\begin{array}{ll} E_{\text{SP}} & g\\ g & E_{\text{m}} \end{array}\right)\left(\begin{array}{l} \alpha_{\text{SP}}\\ \alpha_{\text{m}} \end{array}\right)=E\left(\begin{array}{l} \alpha_{\text{SP}}\\ \alpha_{\text{m}} \end{array}\right)$$

Here, *E*_SP_ and *E*_m_ are the energies of the uncoupled plasmon and monomeric exciton energy, respectively; *g* is the coupling strength between plasmon and monomer exciton. *E* stands for the eigenvalues corresponding to the energies of the hybrid modes. For the value *E*_SP_ of each scattering spectra, it is calculated by $E_{\text{SP}}=E_{\text{HEB}}+E_{\text{LEB}}-E_{\text{m}}$ . The mixing coefficients $\alpha_{\text{SP}}$ and $\alpha_{\text{m}}$ describe the relative plasmon and monomer–exciton weightings of the hybrid modes, which satisfies ${\left|\alpha_{\text{SP}}\right|}^{2}+{\left|\alpha_{\text{m}}\right|}^{2}=1$ . Taking the detuning into consideration, the energy *E* of the hybrid modes can be obtained as:

(2) $$E=\frac{1}{2}\left[E_{\text{SP}}+E_{\text{m}}\pm \sqrt{4g^{2}+\delta^{2}}\right]$$

where $\delta =E_{\text{SP}}-E_{\text{m}}$ is the detuning energy between plasmon and monomer exciton. Thus, the scattering peaks in Figure 3c can then be fitted by Eq. (2). Anti-crossing fitting results are obtained (solid curves), which agrees well with the experimental data. At zero detuning ( $E_{\text{SP}}=E_{\text{m}}$ ), the corresponding splitting energy is ℏΩ_HL_ = 2*g* = 147 meV. This value has to be compared with the plasmon and exciton line widths. The line width of the Ag nanocavity is $\gamma_{\text{SP}}$ = 154 meV (as shown in Figure 2b) and the exciton width is $\gamma_{\text{m}}$ = 90 meV. One thus finds that Rabi splitting energy ℏΩ_HL_ $>\left(\gamma_{\text{SP}}+\gamma_{\text{m}}\right)/2$ , implying that the plasmon-MB monomer exciton interaction satisfies the strong coupling criterion.

**Figure 4:**
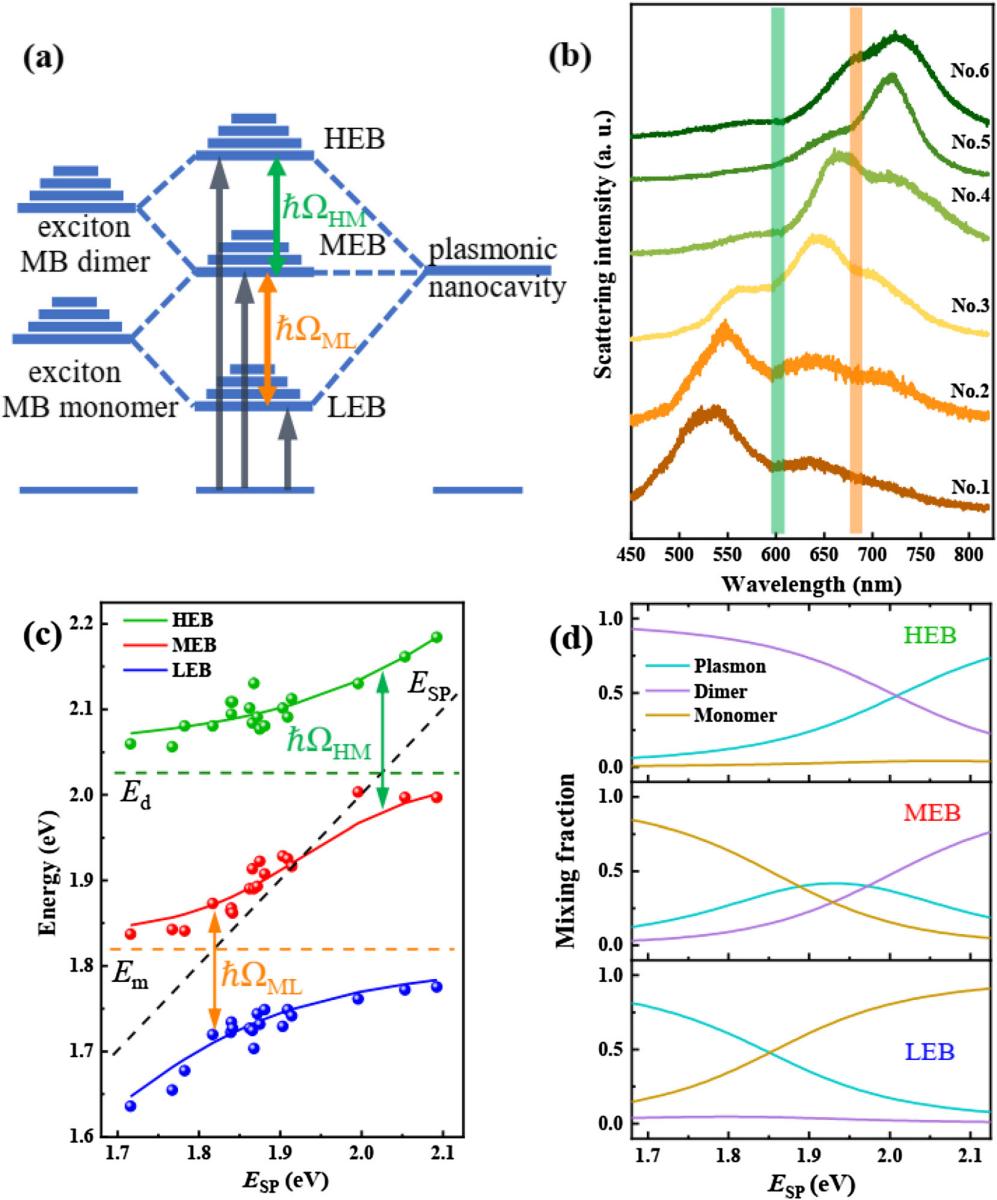
Strong monomer exciton-plasmon-dimer exciton coupling. (a) Schematic representation of the strong coupling between the excitons of the monomeric and dimeric dye molecule (left) and the surface plasmons of nanocavity (right), leading to the formation of three new eigen hybrid light–matter states: HEB, MEB and LEB. The Rabi splitting energies are marked as ℏΩ_HM_ and ℏΩ_ML_, respectively. (b) Scattering spectra of the hybrid nanostructures with different Ag nanocube sizes (sample numbers from No. 1 to No. 6). SPR wavelength of the plasmonic nanocavity is tailored across the exciton resonances of dimer and monomer MB. (c) Dispersion of plexciton with HEB, MEB and LEB varied as a function of plasmon energy. Green, red and blue dots indicate experimental data extracted from scattering spectra. The solid curves are fits of the three-coupled harmonic oscillator model. The black dash line presents the plasmon energy of the pure plasmonic nanocavity, while green and orange dash lines indicate the exciton resonance of MB dimer and monomer. Arrows mark the double Rabi splitting energy of the hybrid nanostructures, giving a splitting of ℏΩ_HM_ = 178 meV and ℏΩ_ML_ = 170 meV. (d) Hopfield coefficients for plasmon, dimer exciton, and monomer exciton contributing to high, middle, and low energy branches (HEB, MEB and LEB) as a function of plasmon energy, calculated using the three-coupled oscillator model.

After the discussion of the monomer exciton–plasmon coupling, we further studied the monomer exciton–plasmon–dimer exciton coupling in the same hybrids. With the increase of dye concentration more dimers are formed. Then the absorbance band of dimer exciton becomes stronger. Thus, the interaction between dimer exciton and plasmon cannot be ignored. Consequently, the hybrid dispersive polariton bands would be produced, as schematically sketched in Figure 4a. Obviously, in the case of a system with a double Rabi splitting, the energy state diagram is somewhat more complicated. Excitons of both the monomer and dimer interact with the plasmons, which leads to three energy branches, marked as HEB, middle energy branch (MEB) and LEB. The Rabi-splitting energies among three branches are denoted as ℏΩ_HM_ and ℏΩ_ML_. To verify the dimer effect on the strong coupling behavior, a high concentration of MB dye (1 × 10^−4^ M) doped PVA layer is spin-coated onto the Ag film. Figure 4b shows the scattering spectra of hybrid nanostructures with different sizes of Ag nanocubes (see more spectra in Figure S3). For each spectrum, there are three peaks, and each peak is red-shifted as the nanocube size increases. While the two dips of scattering spectra are fixed, corresponding to the exciton positions of monomer (orange line) and dimer (green line). It reveals that the interaction among monomer and dimer excitons and plasmon leads to the three hybridized plexciton states, HEB, MEB and LEB.

To map the dispersion curves, we extract the three peaks of multiple scattering spectra, as shown in Figure 4c (green, red and blue dots). The dashed lines indicate the energy positions of plasmons and excitons of monomer and dimer. The traces of all peaks present double anti-crossing curves, which is a typical characteristic of the multi-mode strong coupling among excitons and plasmons [26, 27, 30, 34, 44]. This hybrid coupling system can be approximately described by a three-coupled harmonic oscillator model as [30, 33, 42, 44, 45]:

(3) $$\left(\begin{array}{lll} E_{\text{SP}} & g_{\text{d}} & g_{\text{m}}\\ g_{\text{d}} & E_{\text{d}} & 0\\ g_{\text{m}} & 0 & E_{\text{m}} \end{array}\right)\left(\begin{array}{l} \alpha_{\text{SP}}\\ \alpha_{\text{d}}\\ \alpha_{\text{m}} \end{array}\right)=E\left(\begin{array}{l} \alpha_{\text{SP}}\\ \alpha_{\text{d}}\\ \alpha_{\text{m}} \end{array}\right)$$

where *E*_SP_, *E*_d_ and *E*_m_ are the energies of plasmon, MB dimer and monomer exciton, respectively. $g_{\text{d}}$ and $g_{\text{m}}$ are the coupling strength between plasmon and monomer exciton, and between plasmon and dimer exciton, respectively. *E* represents the eigenvalues corresponding to the energies of the plexciton states. *α*_SP_, *α*_d_ and *α*_m_ are the eigenvector components. The eigenvalues and eigenvectors of Eqs. (1) and (3) can be derived using MATLAB’s eigen function. The corresponding ${\left|\alpha_{\text{SP}}\right|}^{2}$ , ${\left|\alpha_{\text{d}}\right|}^{2}$ and ${\left|\alpha_{\text{m}}\right|}^{2}$ represent the weighting efficiencies (known as the Hopfield coefficients) of plasmon, dimer and monomer excitons in plexciton states, respectively. The coefficients satisfy ${\left|\alpha_{\text{SP}}\right|}^{2}+{\left|\alpha_{\text{d}}\right|}^{2}+{\left|\alpha_{\text{m}}\right|}^{2}=1$ . There are three unique solutions for E ( $E_{\text{H}}$ , $E_{\text{M}}$ and $E_{\text{L}}$ ). Thus, the plexciton states comprise three anti-crossed bands, corresponding to HEB, MEB and LEB (as shown in Figure 4a). For the value $E_{\text{SP}}$ of each scattering spectra, it can be calculated by $E_{\text{SP}}+E_{\text{d}}+E_{\text{m}}=E_{\text{H}}+E_{\text{M}}+E_{\text{L}}$ [42]. Anti-cross fitting results by Eq. (3) are shown in Figure 4c (solid curves), which agree well with the experimental data. On the basis of the fitting, the corresponding coupling strengths are $g_{\text{d}}$ = 89 meV and $g_{\text{m}}$ = 85 meV, respectively. Therefore, the splittings extracted at zero detuning between plasmon and dimer exciton ( $\delta =E_{\text{SP}}-E_{d}=0\text{ eV}$ ) and between plasmon and monomer exciton ( $\delta =E_{\text{SP}}-E_{\text{m}}=0\text{ eV}$ ) respectively are ℏΩ_HM_ = 178 meV and ℏΩ_ML_ = 170 meV. Thus, the three plexciton branches (HEB, MEB and LEB) cover a broad energy range (about 1.7–2.1 eV). Note that no simple criteria for the strong coupling in the three-coupled harmonic oscillator model. Figure 4d shows the Hopfield coefficients for each of the three branches. Specifically, the monomer exciton makes no contribution to the HEB while the dimer exciton makes no contribution to the LEB. At the crossing points of mixing fraction in the HEB and LEB, the hybrid modes are half plasmonic and half excitonic in nature. Interestingly, the MEB contains nonnegligible contributions from both the dimmer and monomer excitons (28% each at their intersection), indicating that the excitons and plasmon are coherently hybridized.

Compared with the splitting energy (147 meV) between monomer exciton and plasmon at low dye concentration, these splitting energies are a little larger. It implies that the MEB shows less disperse [44]. The hybrid system provides a good platform for studying multi-mode strong coupling at nanoscale. These values of Rabi splitting for hybrid structures are comparable to the data reported for two different J-aggregated molecular dyes incorporated in a microcavity [31]. More generally, our findings demonstrate the strong dimer exciton–plasmon coupling at high dye concentration. It suggests that the dye concentration is limited to enhance the coupling strength by increasing the dye concentration. In general, the extent of dye aggregation depends reciprocally on the temperature of the solution and is fully reversible [38]. Considering the reversible conversion between monomer and dimer under different temperatures, the single Rabi splitting and double one can be controlled by temperature in future.

## Conclusions

In summary, we experimentally demonstrated the strong coupling between excitons of MB dye at different concentrations and plasmons in Ag nanocavity. At a low dye concentration, a single Rabi splitting was observed up to 147 meV, which is attributed to the strong coupling between the monomer exciton and plasmon. While the dye concentration is increased, a double Rabi splitting was obtained up to 178 and 170 meV, leading to three new hybridized plexciton modes. It is ascribed to the collective interaction between both monomer and dimer excitons and plasmons, forming the exciton–plasmon–exciton coupling behavior. Double anti-crossing behavior is further presented by measuring the scattering spectra from different Ag nanocube sizes, which is in excellent agreement with the typical signature of strong coupling. The results indicate that the equilibrium of monomer and dimer of MB dye has a significant effect on the coupling behavior. Such a double Rabi splitting significantly expands the region of energy splitting between the plexcitons states.

## Experimental section

**Sample preparation.** The hybrid nanostructures were prepared by a bottom-up nano-assembly technique. First, the silicon wafer is coated with a 10/80-nm-thick Ti/Ag film by E-beam evaporation. Then the mixed solution of PVA-MB was spin-coated on the prepared Ag film at a speed of 1800 rpm. PVA-MB mixed solution is composed of PVA aqueous solution (3%) and MB aqueous solution with a volume ratio of 1:1. PVA is added to increase the adhesive force of the solution and ensure the good quality of the MB gap layer. Finally, the 65–95 nm Ag nanocubes with a 1–3 nm-thick polyvinylpyrrolidone layer (nanoComposix, diluted to 0.01 mg/mL) were dropped onto the prepared polydimethylsiloxane (PDMS). After drying, the PDMS with Ag nanocubes was glued to the Ag film with MB and gently pressed. After 3 min, the PDMS was removed and a hybrid nanostructure was formed. To avoid coupling between Ag nanocubes, Ag nanocubes must be evenly distributed on the Ag film. Note that PDMS was used to transfer the Ag nanocubes to the MB layer to prevent ethanol in the Ag nanocubes solution from damaging the MB layer. The prepared samples are clear and free of impurities, which is an important guarantee for scattering measurement.

**Numerical simulation.** Numerical calculations of NPoM nanocavity were performed using a commercial FDTD solver (Lumerical, Inc.). To obtain the electric field in Figure 2c, an Ag nanocube is positioned above an 80 nm Ag film coated with PVA layer. The edges and corners of Ag nanocubes are rounded by 10 nm according to the TEM images. The dielectric function for Ag was taken from Johnson and Christy [46]. To simulate electric field distribution, the NPoM nanocavity is illuminated vertically by a plane wave. Three monitors (frequency domain power monitors) are placed correspondingly to obtain the electric field maps.

## Supplementary Material

Supplementary Material
