# Supplementary material for: Double Rabi splitting in methylene blue dye-Ag nanocavity
Source: Nanophotonics. 2022 Jan 4;11(3):603–11. doi: 10.1515/nanoph-2021-0697 (PMC11501508; doi:10.1515/nanoph-2021-0697)
Supplement: Supplementary file 1 — Supplementary Material [file j_nanoph-2021-0697_suppl.docx]

**Double Rabi Splitting in** **Methylene Blue Dye-Ag Nanocavity**

Xiaobo Han^1^, Fang Li^1,*^, Zhicong He^1^, Yahui Liu^1^, Huatian Hu^1^, Kai Wang^2,*^, and Peixiang Lu^1,2,3^

^1^Hubei Key Laboratory of Optical Information and Pattern Recognition, Wuhan Institute of Technology, Wuhan 430205, China

^2^Wuhan National Laboratory for Optoelectronics and School of Physics, Huazhong University of Science and Technology, Wuhan 430074, China

^3^Guangdong Intelligent Robotics Institute, Dongguan 523808, China

*Corresponding authors: lifang@wit.edu.cn (FL), kale_wong@hust.edu.cn (KW)


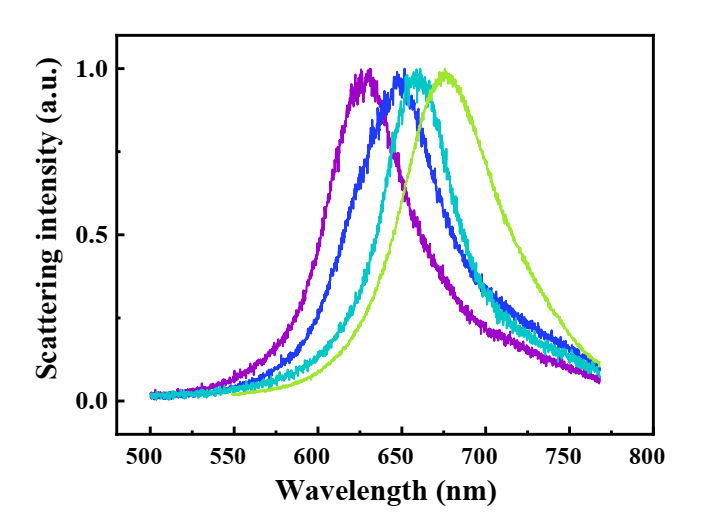


**Figure S1.** Scattering spectra of Ag nanocavities by varying the size of nanocubes (65-95 nm).


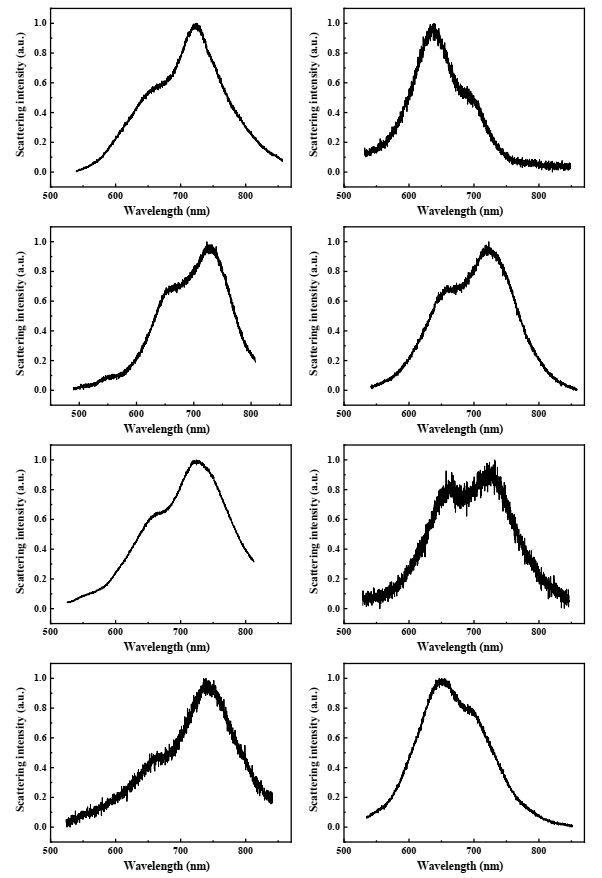


**Figure S2.** Representative scattering spectra of hybrids at a low dye concentration.


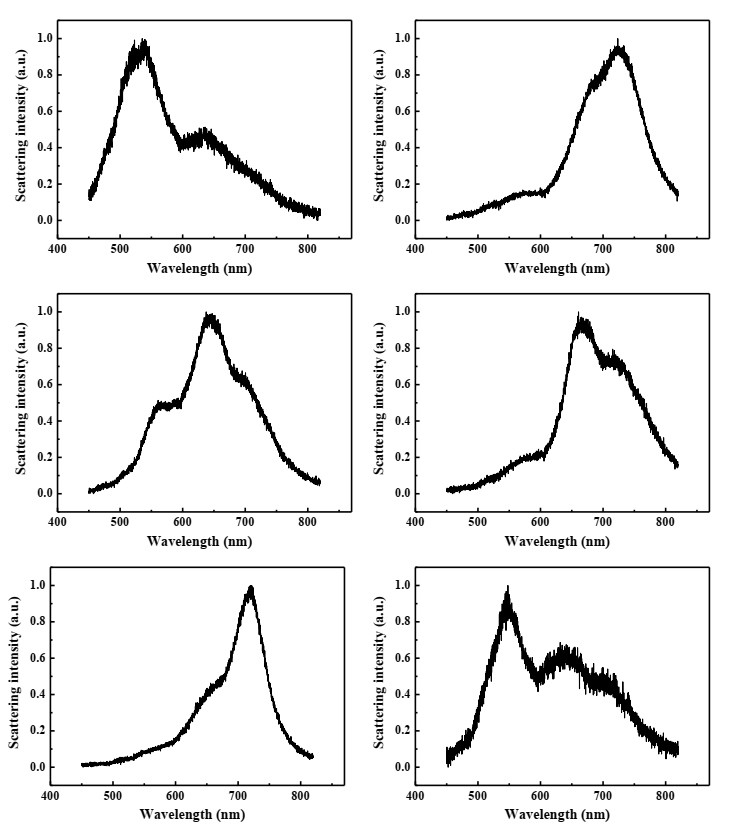


**Figure S3.** Representative scattering spectra of hybrids at a high dye concentration.
